# The Missouri Medical and Surgical Journal

**Published:** 1845-07

**Authors:** 


THE MISSOURI MEDICAL AND SURGICAL JOURNAL, edited by R. F. Stevens, M. D., and published at St. Louis, Mo. The first number of this new auxiliary was issued on the First of May, and the publication is to continue regularly on the first of every month, each number containing "twenty-four pages, closely printed." "The subscription will be two dollars per year, payable always in advance." The number before us contains several original articles by physicians of St. Louis, beside selected matter, forming altogether a respectable journal.

